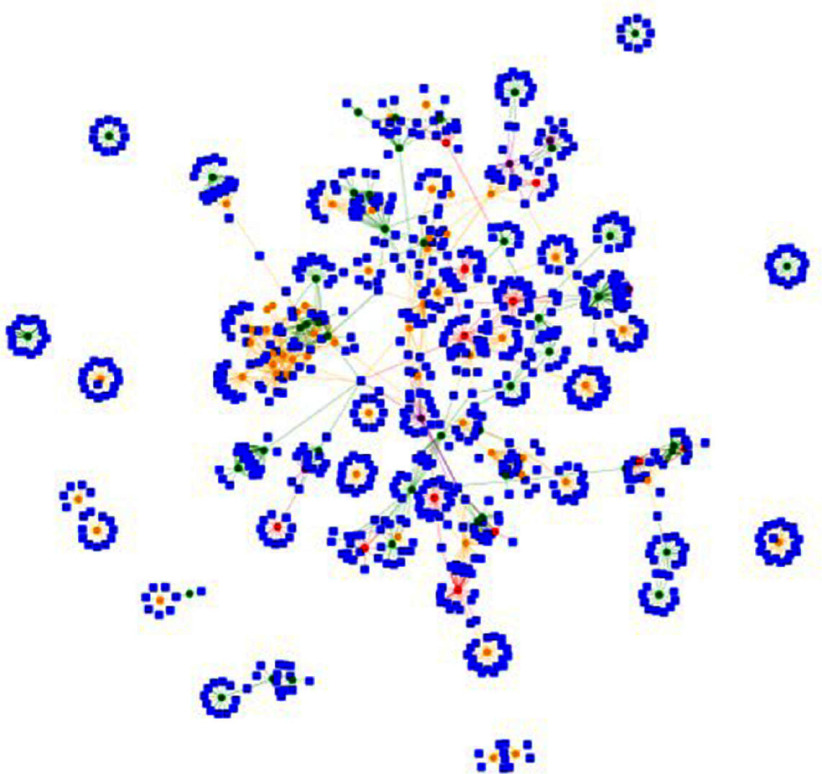# Network Mapping for Vancomycin-resistant Enterococcus (VRE) to Understand Patient-Staff Interactions - A Pilot Study

**DOI:** 10.1017/ash.2025.421

**Published:** 2025-09-24

**Authors:** Fadilah Zakaria, Edwin Philip Conceicao, Indumathi Venkatachalam, Aung Myat Oo, Xiang Ying Jean Sim

**Affiliations:** 1 National University of Singapore; 2 Singapore General Hospital;Weien Chow,; 3 Singapore General Hospital; 4 Singhealth

## Abstract

**Background:** Vancomycin-resistant Enterococcus (VRE) is a pathogen that can cause nosocomial infection leading to increased morbidity and mortality. Healthcare provider interactions have been reported to cause nosocomial infection acquisition. Here we aim to understand patient-staff interactions by mapping their interactions together with the movement of VRE colonised patients in an acute inpatient setting in a tertiary hospital for one month to create a dynamic visualisation map. **Methods:** Staff-patient interactions were obtained through documentation in the electronic health records (EHR). Hospital-onset VRE Hospital-Onset acquisition is defined as a positive screen/culture on or after the third day of admission. The cohort was categorised by their VRE statuses such as Hospital-onset (HO), Community-onset (CO) defined occurring before the 3rd day of admission, Infectious period (XO) and Negative (NO). XO is estimated to be the two days before a positive test HO acquisition. NO patients in this cohort eventually turn positive during their admission. A network was generated where patients and staff were represented as nodes, with edges weighted by number of interactions with the specific staff. Node colours were assigned based on VRE status. An interactive patient-staff interaction network map was developed using Python 3.12.8, using PyVis library version 0.3.2 for network visualization and Dash version 2.18.2 for web-based interactivity. **Results:** There are a total of 207 patients who tested positive for VRE in SGH in July 2024. 54 (26.1%) were HO. A snapshot of the map filtered for 10/07/24 to 11/07/24 can be seen in Figure 1. 14 (11.8%) were HO, 58 (48.7%) were CO, 44 (37.0%) were NO and 3 (2.5%) were XO. As seen in figure 1, there are 7 (5.9%) are in isolated singular clusters, 6 (5.0%) are in pair clusters and 3 (2.5%) are in a triple cluster. In total, the largest cluster consists of 103 (86.6%) patients. This cluster consists of HO,NO,CO and XO patients. This intermingling highlights potential routes for cross-transmission of VRE. **Conclusion:** The network map reveals notable intermingling of CO, HO, XO and NO contacts within the dominant cluster and suggests potential routes of transmission. This underscores the need for better understanding of transmission dynamics to allow enhancement of existing infection prevention policies to prevent this spread.

**Figure:**